# Leader Narcissism and Outcomes in Organizations: A Review at Multiple Levels of Analysis and Implications for Future Research

**DOI:** 10.3389/fpsyg.2017.00773

**Published:** 2017-05-19

**Authors:** Susanne Braun

**Affiliations:** ^1^Durham University Business School, Durham UniversityDurham, United Kingdom; ^2^Center for Leadership and People Management, Ludwig Maximilian University of MunichMunich, Germany

**Keywords:** CEO, dark triad of personality, leadership, levels of analysis, narcissism, organization, team

## Abstract

Narcissists often pursue leadership and are selected for leadership positions by others. At the same time, they act in their own best interest, putting the needs and interests of others at risk. While theoretical arguments clearly link narcissism and leadership, the question whether leader narcissism is good or bad for organizations and their members remains unanswered. Narcissism seems to have two sides, a bright and a dark one. This systematic literature review seeks to contribute to the ongoing academic discussion about the positive or negative impact of leader narcissism in organizations. Forty-five original research articles were categorized according to outcomes at three levels of analysis: the dyadic level (focusing on leader-follower relationships), the team level (focusing on work teams and small groups), and the organizational level. On this basis, we first summarized the current state of knowledge about the impact that leader narcissism has on outcomes at different levels of analysis. Next, we revealed similarities and contradictions between research findings within and across levels of analysis, highlighting persistent inconsistencies concerning the question whether leader narcissism has positive or negative consequences. Finally, we outlined theoretical and methodological implications for future studies of leader narcissism. This multi-level perspective ascertains a new, systematic view of leader narcissism and its consequences for organizations and their stakeholders. The article demonstrates the need for future research in the field of leader narcissism and opens up new avenues for inquiry.

## Introduction

“Half the harm that is done in this world is due to people who want to feel important. They don't mean to do harm, but the harm does not interest them. Or they do not see it, or they justify it because they are absorbed in the endless struggle to think well of themselves.”

— T. S. Eliot, The Cocktail Party (1949).

Current socio-historic developments spur increasing public as well as academic interest in narcissism, especially in relation to leadership (Rosenthal and Pittinsky, 2006; Campbell et al., 2011; Grijalva and Harms, 2014). Statistics suggest that narcissism is particularly prevalent in younger adults today, described as the “Generation Me” (Twenge, 2013). Narcissism seems to touch everyone's lives. New social media such as Facebook, introduced in 2006 with now 1.79 billion active users per month, fuel opportunities for narcissistic self-expression. Self-serving and irresponsible financial practices in the banking sector culminated in a global financial crisis in 2008. And finally, the election of Donald Trump as President of the United States in 2016 has been argued to reflect the far-reaching impact of narcissism on society. Trump is described as “highly extraverted, disagreeable, angry, charismatic, untruthful, and narcissistic” (Visser et al., 2017, p. 281). According to recent research, his public personality profile is suggestive of psychopathy and narcissism triggered by perceptions of exceptionally low humility and agreeableness, very low emotionality and low conscientiousness (Visser et al., 2017). Analyses of his communication profile suggest that Trump has a strong self-promotional style that underscores narcissistic grandiosity (Ahmadian et al., 2017).

While narcissism is a very timely topic to consider, the psychological study of narcissism dates back to more than one century ago. In his seminal essay “On narcissism: An introduction” (1957/1914) Freud first discussed the concept of primary or non-clinical narcissism existing in all individuals, albeit to a certain extent. He distinguished this excessive self-love from clinical disorders. The distinction between Narcissistic Personality Disorder and narcissism as a subclinical personality trait persists to date (Braun et al., 2016; Miller et al., 2017).

The so-called dark triad of personality consisting of Machiavellianism, Psychopathy, and Narcissism belongs to the best-researched concepts of the dark side in organizations to date (O'Boyle et al., 2012). While individuals with these traits generally lack agreeableness in social interactions, they are not equivalent to each other (Paulhus and Williams, 2002). Machiavellianism is characterized by manipulative actions and a lack of concern for others. Psychopathy reflects a combination of thrill seeking and lack of remorse. Narcissism, however, appears to be primarily driven by self-enhancement tendencies. According to Morf and Rhodewalt (2001) narcissists' “grandiose yet vulnerable self-concept appears to underlie the chronic goal of obtaining continuous external self-affirmation” (p. 177). Herein lies the paradox of narcissism; narcissists critically rely on affirmative relationships with their environment, but lack the concern for others in order to maintain positive social relationships in the long run. In other words, narcissists may not intend to harm others, but they are oblivious to others' wellbeing as long as their own needs for self-affirmation and external validation are fulfilled.

Researchers in the field of leadership have been particularly concerned with the paradox of narcissism as well as its potentially detrimental consequences. Earlier theoretical work suggests clear interrelations between narcissism and leadership (Kets De Vries and Miller, 1984; Rosenthal and Pittinsky, 2006), but subsequent empirical results remain mixed with a range of findings from positive to negative or null relationships (Campbell et al., 2011). Meta-analytic results at the dyadic level of analysis found positive relationships between leader narcissism and leader emergence explained by leader extraversion, but indicated that leader narcissism related positively only to self-rated effectiveness (Grijalva et al., 2015). Subsequent results suggested that the relationship between leader narcissism and leader effectiveness rated by others was best described through a curvilinear function (i.e., an optimal midrange of narcissism; Grijalva et al., 2015). Recent evidence supported the notion of deteriorating relationships between narcissistic leaders and their followers over time (Ong et al., 2016). In sum, the question persists: “Is narcissism good or bad?” (Campbell et al., 2011, p. 272).

The question whether narcissism is good or bad is of particular relevance in organizational contexts. Narcissists actively pursue leadership positions and are likely to be selected for them by others, but at the same time tend to act in their own best interest, while putting the needs and interests of others at risk. Campbell et al. (2011) described this as the two sides of narcissism, a bright and a dark one. The bright side occurs when narcissists are at their best, the dark side when they are at their worst. The bright side informs initial impression formation, for example, leaders' charming or visionary attributes. The dark side, such as self-serving and manipulative acts, emerges when narcissists let their guard down. According to Hogan and Kaiser (2005) the two sides “typically coexist with well-developed social skills that mask or compensate for them in the short run. Over time, however, dark side tendencies erode trust and undermine relationships” (p. 171). This systematic literature review seeks to contribute to the ongoing academic debate around the positive or negative impact of narcissism in organizational contexts, with a focus on leaders' narcissism and its outcomes at multiple levels of analysis (i.e., in dyadic relationships of leaders and followers, teams, and the entire organization). More specifically, the main goals of this systematic literature review are threefold (cf. Baumeister and Leary, 1997):

*Firstly*, we aim at systematically analyzing and presenting the current state of knowledge about the impact that leader narcissism has on outcomes at three levels of analysis: (a) at the dyadic level (focusing on leader-follower dyads), (b) the team level (focusing on work teams or small groups), and (c) the organizational level. This multi-level perspective ascertains new, systematic insights into leader narcissism and its consequences for organizations and their stakeholders. It concurs with state-of-the-science views suggesting that leadership is inherently a multi-level phenomenon in organizations. Hence, differentiations between levels of analysis provide meaningful insights into organizational functioning (Klein et al., 1994, 2001; Klein and Kozlowski, 2000; Yammarino et al., 2005; Kozlowski and Ilgen, 2006). This multi-level view is quite relevant for a better understanding of the outcomes of leader narcissism. Narcissists strive for power and prestige (Hansbrough and Jones, 2015). As they ascertain powerful positions and climb the organizational hierarchy, their influence may “trickle down” from the executive to middle management levels, similar to what has been shown for positive leadership styles (Schaubroeck et al., 2012). Yet, outcomes at different levels of analysis are not necessarily similar in nature. For example, while narcissistic leaders may fail to establish positive relationships with followers in the organization, their visionary skills could convince external stakeholders to invest in the company. These possibilities need careful consideration.

*Secondly*, we aim at systematically uncovering paradoxical findings about the outcomes of leader narcissism in organizations. A systematic review should not only reveal similarities but also apparent contradictions between research findings within and between levels of analysis. The review highlights inconsistencies concerning the question whether leader narcissism is good or bad (Campbell et al., 2011) and has positive or negative consequences for the entire organization or its stakeholders. The purpose of this integration of knowledge is to derive implications for future research (e.g., moderators of the impact of leader narcissism on organizational outcomes).

*Thirdly*, we point to methodological weaknesses in current studies of leader narcissism in organizations. On this basis, the article provides recommendations for alternative methodological approaches (e.g., experiments, case studies, computer simulations) to studying leader narcissism and its outcomes at multiple levels of analysis.

The following parts of the article summarize the most influential definitions of narcissism in the context of leadership and organizational research; detail the methodology used for this systematic literature review; summarize main research findings for each level of analysis; and discuss gaps and contradictions in the research to point out recommendations for future work on leader narcissism in organizations.

## Narcissism defined

The term narcissism originated from Greek mythology. A beautiful young man named Narcissus fell in love with his own reflection whilst gazing into a pool of water. Captured by the sight of his beauty, Narcissus kept admiring his reflection and died in despair when realizing that he would be unable to seduce his own image. Freud (1957/1914) first wrote about the differences between primary, but not clinical narcissism, the extent to which individuals are driven by self-love, and secondary narcissism as a form of personality disorder.

The current scientific study of narcissism mainly draws on views from clinical psychology and psychiatry or personality psychology. The former fields are concerned with Narcissistic Personality Disorder (NPD). The Diagnostic and Statistical Manual of Mental Disorders (DSM-5) diagnoses NPD according to several criteria including a grandiose sense of self-importance; fantasies of unlimited success, power, brilliance, beauty, or ideal love; believes that one is “special” and unique; requiring excessive admiration; sense of entitlement, interpersonally exploitative behavior; lack of empathy; being envious of others or believing that others are envious of oneself; arrogant, haughty behaviors or attitudes.

Definitions of narcissism in organizational psychology draw on the criteria indicated above, but define narcissism as a personality trait rather than a mental illness. It is seen as relatively stable and existing in all individuals, albeit to varying degrees. There are several influential definitions of narcissism in organizational psychology (see Table 1).

**Table 1:** **Overview of influential narcissism definitions**.

**Source**	**Page**	**Definition**
Kets De Vries and Miller, 1985	p. 588	“Narcissists feel they must rely on themselves rather than on others for the gratification of life's needs. They live with the assumption that they cannot reliably depend on anyone's love or loyalty. They pretend to be self-sufficient, but in the depth of their beings they experience a sense of deprivation and emptiness. To cope with these feelings and, perhaps, as a cover for their insecurity, narcissists become preoccupied with establishing their adequacy, power, beauty, status, prestige, and superiority. At the same time, narcissists expect others to accept the high esteem in which they hold themselves, and to cater to their needs. What is striking in the behavior of these people is their interpersonal exploitativeness. Narcissists live under the illusion that they are entitled to be served, that their own wishes take precedence over those of others. They think that they deserve special consideration in life.”
Emmons, 1987	p. 15	“Narcissism, rather than being a unidimensional construct, consists of four moderately correlated factors tapping the domains of leadership, self-admiration, superiority, and interpersonal exploitiveness. Only the Exploitiveness/Entitlement subscale was found to correlate significantly with two measures of pathological narcissism. This finding supports previous claims that this factor represents the maladaptive aspects of the trait, indicating that interpersonal maneuvers may be especially troublesome for narcissistic individuals.”
Maccoby, 2000	p. 70	“Leaders such as Jack Welch and George Soros are examples of productive narcissists. They are gifted and creative strategists who see the big picture and find meaning in the risky challenge of changing the world and leaving behind a legacy. Indeed, one reason we look to productive narcissists in times of great transition is that they have the audacity to push through the massive transformations that society periodically undertakes. Productive narcissists are not only risk takers willing to get the job done but also charmers who can convert the masses with their rhetoric. The danger is that narcissism can turn unproductive when, lacking self-knowledge and restraining anchors, narcissists become unrealistic dreamers. They nurture grand schemes and harbor the illusion that only circumstances or enemies block their success. This tendency toward grandiosity and distrust is the Achilles' heel of narcissists. Because of it, even brilliant narcissists can come under suspicion for self-involvement, unpredictability, and—in extreme cases—paranoia.”
Morf and Rhodewalt, 2001	p. 178	“We argue that underlying narcissistic self-regulation is a grandiose, yet vulnerable self-concept. This fragility drives narcissists to seek continuous external self-affirmation. Furthermore, much of this self-construction effort takes place in the social arena. Yet, because narcissists are characteristically insensitive to others' concerns and social constraints, and often take an adversarial view of others, their self-construction attempts often misfire. Thus, although narcissistic strategic efforts generally help maintain self-esteem and affect short term, they negatively influence their interpersonal relationships and in the long run ironically undermine the self they are trying to build. The result is a chronic state of self-under-construction, which they relentlessly pursue through various social-cognitive-affective self-regulatory mechanisms in not always optimal ways.”
Rosenthal and Pittinsky, 2006	p. 629	“Narcissistic leadership occurs when leaders' actions are principally motivated by their own egomaniacal needs and beliefs, superseding the needs and interests of the constituents and institutions they lead. We define egomaniacal needs and beliefs to include many of the patterns pervasive in narcissistic personality—grandiose sense of self-importance, preoccupation with fantasies of unlimited success and power, excessive need for admiration, entitlement, lack of empathy, envy, inferiority, and hypersensitivity (American Psychiatric Association, 2000). What is critical about this definition, and what differentiates it from simply describing narcissistic leaders, is that it is sensitive to the context in which the leadership takes place—as with theories of power motivation, narcissistic leadership considers leaders' psychological motivations; and as with charismatic leadership, narcissistic leadership takes situational factors and follower perceptions into account. Unlike the study of narcissistic leaders, it is not directly linked to leader personality traits, including their narcissism—non-narcissists can engage in narcissistic leadership, whereas narcissists are capable of leading non-narcissistically.”
Campbell et al., 2011	p. 269	“Narcissism is a relatively stable individual difference consisting of grandiosity, self-love and inflated self-views (For reviews see Morf and Rhodewalt, 2001; Campbell et al., 2006). It is useful to think of narcissism as containing three components: the self, interpersonal relationships and self-regulatory strategies. First, the narcissistic self is characterized by positivity, “specialness” and uniqueness, vanity, a sense of entitlement and a desire for power and esteem. Second, narcissistic relationships contain low levels of empathy and emotional intimacy. In their place, there are (often numerous) shallow relationships that can range from exciting and engaging to manipulative and exploitative. Third, there are narcissistic strategies for maintaining inflated self-views. For example, narcissists seek out opportunities for attention and admiration, brag, steal credit from others, and play games in relationship. When narcissists are successful at this, they feel good—they report high self-esteem and positive life satisfaction (Sedikides et al., 2004). When they are unsuccessful, they evidence aggression and sometimes anxiety and depression (Bushman and Baumeister, 1998; Miller et al., 2007).”
Pincus et al., 2014	pp. 439f.	“To the layperson, narcissism is most often associated with arrogant, conceited, and domineering attitudes and behaviors, which are captured by the term narcissistic grandiosity. This accurately identifies some common expressions of maladaptive self-enhancement, disagreeableness, and lack of empathy associated with pathological narcissism. However, an emerging contemporary clinical model of pathological narcissism (Pincus and Lukowitsky, 2010; Roche et al., 2013) combines grandiosity with clinically important regulatory impairments that lead to self, emotional, and behavioral dysregulation in response to ego threats or self-enhancement failures (see Figure 1).”

A seminal theoretical piece by Kets De Vries and Miller (1985) linked narcissism and leadership. It suggested that an underlying narcissistic personality dimension is prevalent in most leaders, and that the nature and degree of narcissism reflect in leaders' behaviors. This characterization of narcissism also pointed to its paradox, namely that narcissists “live with the assumption that they cannot reliably depend on anyone's love or loyalty” but at the same time need others to confirm their sense of “adequacy, power, beauty, status, prestige, and superiority” (Kets De Vries and Miller, 1985, p. 588). These authors also highlighted narcissists' tendencies to engage in interpersonal exploitation and their sense of entitlement.

Early empirical work by Emmons (1984, 1987) as well as Raskin and Hall (1979), Raskin and Hall (1981), and Raskin and Terry (1988) clearly inspired narcissism research in the field of leadership. This work targeted the multidimensional nature of narcissism and its measurement. Development and validation of the Narcissistic Personality Inventory (NPI) resulted in a four-dimensional conceptualization of narcissism, including Exploitiveness/Entitlement (feeling entitled to special treatment even at the expense of others' needs and interests); Leadership/Authority (striving to exert influence over others and belief in one's superior leadership qualities); Superiority/Arrogance (feeling better than others); Self-absorption/Self-admiration (seeing oneself as special). This conceptualization continues to influence measurement approaches of leader narcissism to date.

The close links between leadership and narcissism have also been strengthened by practice-oriented work. Maccoby (2000) argued that while “most people think of narcissists in a primarily negative way […] narcissism can be extraordinarily useful—even necessary” (p. 70). Distinguishing between productive and unproductive narcissism, this work described productive narcissists as “risk takers willing to get the job done but also charmers who can convert the masses with their rhetoric” (Maccoby, 2000, p. 70). Unproductive narcissists were depicted as “unrealistic dreamers” who overestimate their own capabilities and discount advise from others (Maccoby, 2000, p. 70). The strengths of narcissistic leaders hence lie in their visionary qualities, yet lacking the skills to collaborate. This view corresponds to assumptions about dark and bright sides of leader narcissism (Hogan and Kaiser, 2005).

Later theoretical views advanced the understanding of narcissism as an interpersonal dynamic. The dynamic self-regulatory model of narcissism (Morf and Rhodewalt, 2001) suggested that narcissists are driven by “a grandiose, yet vulnerable self-concept” (p. 178). That is, narcissists hold strong, over-idealized self-views that require constant affirmation from others, also described as “a chronic state of self-under-construction” (Morf and Rhodewalt, 2001, p. 178). Again, this definition illustrates the paradox of narcissism. Narcissistic striving for self-affirmation, regardless or even at the cost of others' needs and interests, destroys interpersonal relationships.

Recent work further emphasized the relational nature of narcissism in the form of narcissistic leadership (Rosenthal and Pittinsky, 2006). The authors differentiated leaders' personality traits from their behaviors to suggest that “non-narcissists can engage in narcissistic leadership, whereas narcissists are capable of leading non-narcissistically” (p. 629). While this view deemphasizes the direct impact of personality, it still aligns with Campbell et al. (2011), who synthesized the characteristics of narcissism in organizational contexts to suggest three main components: grandiose sense of one's self; exploitativeness and lack of empathy in relationships; and narcissistic strategies to maintain the over-idealized, but fragile self-views.

Finally, authors in the clinical field differentiated between two phenotypic subtypes, narcissistic grandiosity and narcissistic vulnerability (Pincus et al., 2014). Grandiose narcissists are described as extraverted individuals with at least moderate levels of self-esteem and low neuroticism, whereas vulnerable narcissists suffer from low self-esteem and high neuroticism. Both sub-types are unlikely to display agreeableness or build lasting relationships with others (Miller et al., 2011). Research in organizational contexts, however, primarily focused on grandiose narcissism as it overlaps more clearly with the stereotypical view of “the classic narcissist in the workplace” (Campbell et al., 2011, p. 270).

In sum, we can assert that organizational psychology conceptualizes narcissism as a personality trait entailing a grandiose sense of the self, paired with self-affirmative strategies and disregard for others. The paradox of narcissism lies within this disregard for others, but need for approval from them. The strategies that narcissists use for self-affirmative purposes may harm social relationships, yet this is not necessarily the case. To ascertain the approval of others, narcissists may strive for power, seek to influence others, and engage in creative or risky actions for success. From a theoretical standpoint, leader narcissism holds the potential for positive and negative consequences in organizations, which is the focus of this review.

## Methods

The article entails a systematic literature review. This approach is characterized by an explicitly documented and replicable search of published research as described below. We followed guidelines for systematic literature reviews (Baumeister and Leary, 1997), and best-practice examples from previous reviews of leadership (Gardner et al., 2011), narcissism (Campbell et al., 2011), and other topics in organizational psychology (Posthuma et al., 2002; Braun et al., 2012).

### Literature search

The first step of this systematic literature review was to search EBSCO Host databases for keywords related to leader narcissism and subsequent outcomes in organizations. Keywords included “leader” or “leadership,” “manager,” or “management,” “CEO,” “narcissism,” or “narcissistic,” “employee,” or “follower,” and variations thereof. The literature search yielded publications between 1921 and 2016. To ensure research quality, we focused on publications in scholarly, peer-reviewed journals. These publications have been subject to the peer review process and hence findings from this work will be more likely to be based on sound theory and methodology. Overall, the literature search yielded 121 original articles. Subsequently, three criteria for inclusion of original articles in the review were established as described below.

### Criteria for inclusion

The first criterion for inclusion in this review was that the original article focused on subclinical narcissism, that is, the view of narcissism as a personality trait (Emmons, 1984, 1987; Paulhus and Williams, 2002; Braun et al., 2016). While we acknowledge the importance of clinical as well as psychodynamic and psychoanalytic literatures, they largely center on NPD. Instead, the purpose of our review was to present narcissism research in the tradition of personality and organizational psychology (Campbell et al., 2011).

The second criterion for inclusion was that the original article incorporated at least one form of assessment of leader narcissism. The purposeful inclusion of alternative assessment approaches (e.g., case studies, computer simulations, historiometric data) beyond common quantitative survey measures enriched the range and depth of this review. While many variations were found (e.g., self and other ratings, video based ratings, objective indices), quantitative measures were generally the most frequent approaches to the assessment of leader narcissism.

The third criterion for inclusion was that the original article included at least one form of assessment of an outcome measure. This criterion was necessary as the purpose of the review was to describe relationships between leader narcissism and organizational outcomes. Outcomes spanned three levels of analysis: the dyad (i.e., perceptions, work-related attitudes, behaviors or objective outcomes in leader-follower dyads), the team (i.e., perceptions, work-related attitudes, behaviors, or objective outcomes in work teams or small groups), and the organization (perceptions, work-related attitudes, behaviors, or objective outcomes in organizations).

Based on these three criteria, we narrowed the number of included publications down to 45 original articles. Of these articles, 21 pertained to outcomes at the dyadic level, five to the team level, and 19 to the organizational level.

### Categorization

For each of the included original articles, the following information was assessed: (1) general information (author names, title, year of publication, journal, abstract), (2) level of analysis (dyad, team, organization), (3) study design (field survey, experiment, other), (3) theoretical definition of narcissism, (4) assessment of narcissism, and (5) variables assessed. We used this information to categorize the articles and present their main findings subsequently.

## Results

We begin by summarizing research designs and assessment approaches to leader narcissism, which have been used in the 45 original articles, before providing more detailed results in relation to each of the three levels of analysis.

### Research design and assessment

The majority of original articles (35) included data collected in the field, while six articles employed experimental research designs (Nana et al., 2010; Nevicka et al., 2011a,b, 2013; Braun et al., 2016; Ong et al., 2016). Alternative approaches included case studies (Jones et al., 2004), computer simulations (Chen, 2010a,b), and historiometric data (Deluga, 1997).

Quantitative measures of leader narcissism included self-ratings as well as other-ratings. The majority of self-ratings employed validated scales, most prominently the 40-item Narcissistic Personality Inventory (NPI; Raskin and Terry, 1988), its 16-item version (NPI-16; Ames et al., 2006) or variations thereof. Other measures used included narcissism scales taken from the California Psychological Inventory (CPI; Wink and Gough, 1990), the Dirty Dozen (Jonason and Webster, 2010) and the short Dark Triad (SD3; Jones and Paulhus, 2014).

One frequently used alternative to the above mentioned ratings was an index originally created by Chatterjee and Hambrick (2007). This measure of narcissism occurred exclusively in studies assessing leader narcissism at the organization level, that is, in studies focusing on the impact of CEO's narcissism. The authors developed and validated the index consisting of several ratings: prominence of the CEO's photograph in annual reports and in press releases, the CEO's use of first-person singular pronouns in interviews, and their compensation relative to the second-highest-paid firm executive (Chatterjee and Hambrick, 2007).

Table 2 summarizes the quantitative measures of leader narcissism.

**Table 2:** **Quantitative measures of leader narcissism**.

**Original measure**	**Used in**	**Rating source**
Narcissistic Personality Inventory (NPI); Raskin and Terry, 1988	Popper, 2002; Brunell et al., 2008; Galvin et al., 2010; Nevicka et al., 2011b; Hochwarter and Thompson, 2012; Ong et al., 2016	Self
	*Variation in:* Leising et al., 2013	
	Deluga, 1997	Others
	*Variation in:* Nevicka et al., 2013	
Narcissistic Personality Inventory short (NPI-16); Ames et al., 2006	Nevicka et al., 2011a; Foti et al., 2012; Peterson et al., 2012; Wales et al., 2013; Greaves et al., 2014; Reina et al., 2014; De Hoogh et al., 2015; Owens et al., 2015	Self
	*Variations in:* Braun et al., 2016; Nevicka et al., 2013	
Pathological Narcissism Inventory (PNI), German version; Morf et al., 2016	Leising et al., 2013	Self
California Psychological Inventory (CPI), narcissism scale; Wink and Gough, 1990	Blair et al., 2008	Self
Supernumerary Personality Inventory (SPI), egotism and manipulativeness scales; Paunonen, 2002	Paunonen et al., 2006	Self
Dark Triad, narcissism scale; Jones and Paulhus, 2014	Martin et al., 2016	Self
Hogan Development Survey, “Bold” scale; Hogan and Hogan, 1997	Khoo and Burch, 2008	Self
Dirty Dozen, narcissism scale; Jonason and Webster, 2010	Wisse et al., 2015; Wisse and Sleebos, 2016	Self
	Volmer et al., 2016	Others
Gough Adjective Check List (ACL); Gough and Heilbrun, 1965	Resick et al., 2009; O'Reilly et al., 2014; Braun et al., 2016	Others
Ratio of first-person singular pronouns to total first-person pronouns in CEO interviews; Raskin and Shaw, 1988	Aktas et al., 2016	Others
Prominence of CEO photograph in annual reports, in press releases, use of first-person singular pronouns in interviews, compensation relative to the second-highest-paid firm executive; Chatterjee and Hambrick, 2007	Chatterjee and Hambrick, 2007; Patel and Cooper, 2014; Engelen et al., 2016 *Variations in:* Olsen et al., 2014; Gerstner et al., 2015; Zhu and Chen, 2015; Olsen and Stekelberg, 2016; Oesterle et al., 2016	Others
15 objective indicators: publicity, awards, lines of biography in the Marquis Who's Who data base, corporate jet use, cash compensation, total compensation, ratio cash compensation, ratio total compensation, rank compensation, role duality, role titles, governance index, photograph, value of acquisitions, number of acquisitions	Rijsenbilt and Commandeur, 2013	Others
Video based rating with adaptation of Narcissistic Personality Inventory (NPI)	Petrenko et al., 2016	Others
California Q-set (CAQ) narcissism prototype; willfulness, hypersensitivity, autonomy scales; Wink, 1992	Sosik et al., 2014	Others
2-item adjective scale	Nana et al., 2010	Others
Perceived supervisor narcissism scale	Hochwarter and Thompson, 2012	Others
Balanced Inventory of Desirable Responding (BIDR), impression management and self-deceptive enhancement scales; Paulhus, 1991	Paunonen et al., 2006	Others

### Leader narcissism and outcomes

In the following sections, we summarize the results pertaining to the question how leader narcissism impacts outcomes at three levels of analysis: leader-follower dyads, teams, and organizations. For each original article included in the review, the main research findings and similarities or differences between findings are highlighted. Table 3 summarizes the articles, indicating outcome variables, theoretical predictions, and empirical findings.

**Table 3:** **Summary of the empirical evidence for the outcomes of leader narcissism at dyadic, team, and organizational levels of analysis**.

**Outcome variable**	**Source**	**Predictions**	**Results**
**DYADIC LEVEL OF ANALYSIS**			
**Leadership Perceptions**			
Dominance	Leising et al., 2013	+	✓
Affiliation		−	✓
Leadership perceptions	Paunonen et al., 2006	+ (bright side)	✓
		− (dark side)	(✓)
Attributed leader charisma	Galvin et al., 2010	+	✓
mediated by vision boldness		+	✓
mediated by socialized visions		−	✓
Personalized charismatic leadership	Popper, 2002	+	✓
Socialized charismatic leadership		−	✓
Avoidant attachment		+	✓
Secure attachment		−	✓
Perceived charismatic leadership	Deluga, 1997	+	✗
Perceived presidential performance		+	✓
Perceived transformational leadership	Greaves et al., 2014	−	✓
Leader wisdom		−	✗
Perceived transformational leadership	Judge et al., 2006	+ (self-rating)	✓
		− (other rating)	(✓)
Self-leader profile	Foti et al., 2012		
Narcissistic leader		+	✓
Anti-prototypical leader		+	✓
**Leadership Effectiveness**			
Perceived leader performance	Blair et al., 2008		
Interpersonal performance		−	(✓)
Conceptual performance		−	✗
Integrity		−	(✓)
Perceived leader effectiveness	De Hoogh et al., 2015		
Moderated by leader gender		− (female leaders)	✓
Moderated by follower gender		− (male followers)	✓
Perceived ethical leadership	Hoffman et al., 2013	−	✗
Perceived leadership effectiveness			
Moderated by ethical context		+/−	✓
Perceived desirability	Nevicka et al., 2013		
moderated by contextual uncertainty		+	✗
Perceived manipulativeness		+	✓
Moderated By Contextual Uncertainty		−	✗
Leader preference			
Moderated By Contextual Uncertainty		+	✓
Mediated by uncertainty reduction		+	✓
Perceived leader effectiveness	Nana et al., 2010	+	✓
**Follower Outcomes**			
Follower innovative behavior (idea generation, idea promotion, and idea implementation)	Wisse et al., 2015		
Moderated by leader narcissism		0 (high narcissism)	✓
Follower malicious envy	Braun et al., 2016	+	✓
Follower benign envy		−	(✓)
Follower counterproductive work behavior		+	✓
Perceived leader effectiveness	Martin et al., 2016		
Mediated by task-, relational-, and change-oriented leadership behaviors		−	✓
Follower citizenship behaviors			
Mediated By Task-, Relational-, And Change-Oriented Leadership Behaviors		−	✓
Follower counterproductive behaviors			
Mediated by task-, relational-, and change-oriented leadership behaviors		+	✓
Follower subjective career success	Volmer et al., 2016	+	(✓)
Follower objective career success (salary, promotions)		+	✓
Follower emotional exhaustion		+	✗
Follower job satisfaction		−	✗
Follower psychological empowerment	Sosik et al., 2014	+ (constructive narcissism)	✓
		− (destructive narcissism)	✓
Follower moral identity		+ (constructive narcissism)	✓
		− (destructive narcissism)	✓
Perceived leader effectiveness	Owens et al., 2015		
moderated by leader humility		+ (high humility)	✓
Follower job engagement			
moderated by leader humility		+ (high humility)	✓
Follower subjective performance			
Moderated by leader humility		+ (high humility)	✓
Follower objective performance			
Moderated by leader humility		+ (high humility)	✓
Follower work outcomes	Hochwarter and Thompson, 2012		
Frustration			
Moderated by enactment behavior		+	✓
Tension			
Moderated by enactment behavior		+	✓
Resource availability			
Moderated by enactment behavior		−	✓
Job performance			
Moderated by enactment behavior		− (low enactment)	✓
**TEAM LEVEL OF ANALYSIS**			
Abusive supervision	Wisse and Sleebos, 2016	0	✓
moderated by position power		0	✓
Leadership emergence	Brunell et al., 2008	+	✓
Motivation to lead		+	✓
Individual task performance		+	✗
Leadership emergence	Ong et al., 2016	+	✓
mediated by transformational leadership		+	(✓)
Moderated by time		− (decrease over time)	✓
Leadership emergence	Nevicka et al., 2011a	+	✓
moderated by reward interdependence		+	✗
Individual task performance			
moderated by reward interdependence		+/− (high interdependence)	(✓)
Perceived leadership effectiveness	Nevicka et al., 2011b	+	✓
Mediated by perceived leader authority		+	✓
Team performance		−	✓
Mediated by information exchange		−	✓
**ORGANIZATIONAL LEVEL OF ANALYSIS**			
**CEO Outcomes**			
Executive compensation	O'Reilly et al., 2014		
Compensation packages			
moderated by organizational tenure		+ (longer tenured CEOs)	✓
Shares of focal-company stock			
Moderated by organizational tenure		+ (longer tenured CEOs)	✓
Executive team pay gap			
Moderated by organizational tenure		+ (longer tenured CEOs)	✓
Transformational leadership	Resick et al., 2009	−	✗
Contingent reward leadership		−	✓
Servant leadership	Peterson et al., 2012	−	✓
Mediated by organizational identification		−	✓
**Organizational Strategy and Culture**			
Dynamism of company strategy	Chatterjee and Hambrick, 2007	+	(✓)
Number and size of acquisitions		+	✓
Extreme company performance		+	✓
Fluctuation in company performance		+	(✓)
Company performance at crisis-onset	Patel and Cooper, 2014	−	✓
Post-crisis company performance		+	✓
M&A outcomes	Aktas et al., 2016		
Acquirer initiation		+ (acquiring CEO)	✓
		− (target CEO)	✗
Private process length		− (acquiring CEO)	✓
Bid premium		+ (target CEO)	✗
Acquirer announcement returns		− (target CEO)	✓
Probability of deal completion		− (target CEO)	✓
Target CEO prestigious position		− (acquiring CEO)	✓
		+ (target CEO)	✗
Growth in internationalization	Oesterle et al., 2016	+	✓
High-risk foreign sales		+	✗
Dominant in-group culture	Jones et al., 2004	+	✓
Professional out-group counterculture		+	✓
**Entrepreneurial Orientation**			
Firm performance variance	Wales et al., 2013	+	✓
Mediated By Entrepreneurial Orientation		+	✓
Shareholder value	Engelen et al., 2016	−	✓
Moderated by market concentration		+ (concentrated markets)	✓
Moderated by market dynamism		+ (dynamic markets)	(✓)
New technology adoption	Gerstner et al., 2015	+	✓
Moderated by audience engagement		+	✓
Mediated by managerial attention		+	✓
**Organizational Image**			
Corporate social responsibility	Petrenko et al., 2016	+	✓
Corporate philanthropy media profile		+	✓
Company performance			
Moderated by CEO narcissism		−	✓
Public financial performance	Olsen et al., 2014		
Earnings-per-share		+	✓
Mediated by operational choices		+	✓
Mediated by accounting choices		+	✗
Stock price		+	✓
Corporate tax sheltering	Olsen and Stekelberg, 2016		
Uncertain tax benefits		+	✓
Effective tax rate		−	✓
Fraud accusations	Rijsenbilt and Commandeur, 2013	+	✓
Financial misreporting	Chen, 2010a	+	✓
moderated by CEO dishonesty		+	✓
moderated by shareholder expectations		+	✓
Moderated by media praise		+	✓
Financial misreporting	Chen, 2010b	+	✓
moderated by CEO dishonesty		+	✓
Moderated by shareholder expectations		+	✓
Moderated by media praise		+	✓
Moderated by social constraints		− (decrease with constraints)	✓
**Top Management Team**			
Top management team behavioral integration	Reina et al., 2014		
Moderated by organizational identification		+ (high identification)	✓
		− (low identification)	✓
Company performance			
Moderated by organizational identification		+ (high identification)	✓
		− (low identification)	✓
Risk-taking spending	Zhu and Chen, 2015		
Moderated by narcissism similarity with CEO		+	✓
Moderated by prior experience with CEO narcissism		+	✓

*N = 45 articles. Predictions: + positive relationship, − negative relationship, 0 no relationship. Results: ✓ supported, (✓) partially supported, ✗ not supported. M&A: Mergers and acquisitions*.

#### Dyadic level of analysis

##### Leadership perceptions

The publications, which center on outcomes of leader narcissism at a dyadic level, stand in the tradition of applied social and organizational research. This field has identified manifold outcomes of leadership (Hiller et al., 2011). The first aspect addressed in this review is how followers perceive narcissistic leaders. Building on the notion of bright and dark sides of leader narcissism (Campbell et al., 2011), one main question that these studies seek to answer is whether leader narcissism results in negative or positive follower perceptions or both. They highlight differences between self-ratings and other-ratings of the outcomes of leader narcissism and studies that consider leader narcissism as one unified construct or assess different sub-facets.

Leising et al. (2013) surveyed 129 German university students together with 377 informants, who provided an external evaluation of the target person. The authors found strong positive correlations between participants' self-rated claim to leadership, which overlapped with narcissistic grandiosity, and self-ratings and other-ratings of dominance. In contrast, negative correlations occurred between claim to leadership and affiliation ratings.

Paunonen et al. (2006) differentiated between characteristics that they classified as bright sides (egotism, self-esteem) and dark sides (manipulativeness, impression management) of leader narcissism. While self-ratings of both egotism and self-esteem positively predicted others' leadership perceptions (i.e., being seen as a natural leader), the two dark side characteristics did not display the assumed negative relationships with this outcome. They functioned as suppressor variables: the bright-side characteristics of narcissism only related positively to followers' leadership perceptions, when ratings of the dark-side characteristics were low. Hence, the authors concluded that narcissistic leaders are more likely to be seen positively by their followers when they exhibit a specific profile of high bright-side and low dark-side characteristics.

In contrast, Galvin et al. (2010) analyzed narcissism as a holistic concept rather than separating dark and bright characteristics related to it. They first assessed the relationship between narcissism and ascribed charisma. Next, they tested whether perceptions of vision boldness rather than socialized visions would facilitate positive views of narcissistic leaders. Results supported the indirect relationship between leader narcissism and attributions of charisma. According to this research, narcissistic leaders are seen as charismatic because they appear passionate, daring, willing to take risks, and lacking fear or hesitancy. Yet, their visions lack in collective appeal and consideration of the greater good. Pointing in the same direction, Popper (2002) found that leader narcissism correlated negatively with other-ratings of socialized charismatic leadership and positively with personalized charismatic leadership. Exploring potential reasons behind these links, the author found that narcissistic leaders were more likely to show avoidant attachment patterns than their less narcissistic counterparts. These findings support the notion that narcissistic leaders communicate compelling visions, but struggle to connect to their followers, who would implement these visions in the long run. The findings also align with a study using historiometric procedures to assess perceived narcissism, charismatic leadership (consisting of charisma and creativity perceptions), and performance of 39 American presidents. The five archival measures of rated presidential performance included mean greatness scores, consensus of greatness, war avoidance, war entry, and great decisions cited. Findings confirmed that presidents seen as narcissistic were also rated more highly in charisma and performance (except for the outcome war entry) than their less narcissistic counterparts (Deluga, 1997).

Above and beyond charismatic and visionary qualities, the concept of transformational leadership incorporates care and consideration for others (Bass, 1985; Bass and Avolio, 1994). Three original articles addressed leader narcissism as a predictor of transformational leadership. Greaves et al. (2014) found in a sample of 77 employees of a high school that self-rated narcissism correlated negatively with colleagues' perceptions of transformational leadership. In a sample of 80 New Zealand business leaders and senior managers, scores of the narcissistic sub-dimension “Bold” negatively predicted self-ratings of transformational leadership. However, correlation patterns indicated that while narcissism related negatively to individualized consideration, its relationship with attributed idealized influence was positive. According to these findings, narcissistic leaders may have different sides reflecting their charisma, but also disregard for others. In addition, Judge et al. (2006) collected empirical evidence of the differences between self- and other-ratings of transformational leadership in relation to leader narcissism. While leader narcissism consistently predicted positive perceptions of one's own transformational leadership, results for follower perceptions were mixed: while in one study, narcissistic leaders were seen as transformational (albeit to a lesser degree than in self-ratings), the second study showed negative relationships between leader narcissism and an improved measure of transformational leadership.

Finally, Foti et al. (2012) used a new, pattern oriented approach to leadership perceptions. They surveyed 491 college students with initial leadership experience regarding their perceptions of self and ideal leadership as well as self-ratings of narcissism and leadership self-efficacy. The authors examined narcissism together with participant gender and leadership self-efficacy as links between self and ideal leader profiles. All three variables, including narcissism, were directly related to participants' self-as-leader profiles. Specifically, narcissistic individuals were more likely to describe themselves as narcissistic leaders (i.e., higher than average responses to intelligence, dedication, and tyranny with average responses to sensitivity) and anti-prototypical leaders (i.e., lower than average responses to sensitivity, intelligence, and dedication with higher than average responses to tyranny). These findings complement the above results, indicating that narcissists endorse views of themselves that include negative or undesirable qualities.

In sum, findings from these nine original articles suggest that while narcissists tend to self-ascribe positive leadership qualities, others do not consistently see these qualities in them. At the same time, narcissists also endorsed self-views that included negative leadership characteristics. The visionary boldness is one reason why narcissists are perceived as leaders, which can be seen as their bright side. Yet, narcissists also dominate others rather than to affiliate with them.

##### Leadership effectiveness

The criterion of leadership effectiveness goes above and beyond perceptions of narcissists as leaders *per se*. It concerns the question whether narcissistic leaders are seen as making a positive impact. Studies including effectiveness criteria point to differences between rating sources and highlight moderators such as leader gender and contextual factors (e.g., ethics, uncertainty). The results also point to differences between leadership effectiveness criteria.

Blair et al. (2008) studied leaders' interpersonal performance (i.e., participation, confrontation effectiveness, team building, sensitivity), conceptual performance (i.e., analysis, judgment and decision making, planning and organizing, initiative), and integrity. They collected data from 154 managers, their immediate supervisor and subordinates. While leader narcissism related negatively only to supervisory ratings of leaders' interpersonal performance and integrity, surprisingly, leader narcissism did not relate significantly to conceptual performance outcomes, independent of the rating source. These findings suggest that supervisors and subordinates have different views of the effectiveness of narcissistic leaders.

In relation to moderating variables on the leader's side, De Hoogh et al. (2015) examined the relationship between leader narcissism and perceived leader effectiveness in 145 leader-follower dyads from a gender perspective. Narcissism violates gendered expectations for women to display communal qualities. Therefore, the authors hypothesized that only narcissistic female leaders would be seen as lacking leadership effectiveness. Indeed, leader narcissism was negatively related to perceptions of leader effectiveness for female leaders, but not for male leaders. Also followers' gender played a role in this relationship. Narcissistic women in leadership positions were viewed as particularly ineffective by male followers. Initial empirical findings suggest further that perceivers use leaders' facial features to draw conclusions about their narcissism and leadership effectiveness. In a laboratory experiment, male leaders were generally perceived as more effective than female leaders. In this study, however, perceptions of leader narcissism related positively to perceived leadership effectiveness (Nana et al., 2010).

Also the organizational contexts, in which narcissistic leaders operate, can alter leadership effectiveness perceptions. Hoffman et al. (2013) assumed that when ethical contexts stand in contrast to narcissistic leaders' actions, they should be seen as unethical and ineffective. In a sample of 68 managers, no direct relationships between self-ratings of leader narcissism and followers' perceptions of ethical leadership or leadership effectiveness were found. However, for followers in organizational contexts perceived as ethical, leader narcissism related significantly negatively to their perceptions of ethical leadership and leadership effectiveness. No significant relationships were obtained in unethical contexts. Hence, a misfit to the organizational context seemed to drive followers' negative evaluations of narcissistic leaders' effectiveness. In contrast, findings from three experimental studies by Nevicka et al. (2013), suggest that contextual conditions can also improve the effectiveness of narcissistic leaders. In uncertain contexts, participants found leader narcissism more desirable and preferred leaders with higher narcissism levels. This positive effect of leader narcissism was mediated by participants' assumptions that selecting this type of leader would reduce their felt uncertainty.

Overall, findings from five original articles suggest that leader narcissism does not necessarily harm perceptions of leadership effectiveness. Rather, it depends on the rating source (supervisor, subordinate), the effectiveness criterion, contextual factors (e.g., ethical culture) and individual difference factors (e.g., leader gender) whether narcissistic leaders are seen as effective.

##### Follower outcomes

The subsequently presented studies analyzed how leader narcissism related to outcomes on the side of their followers. While the majority of findings point to negative effects (e.g., undermining perceived innovative behavior, eliciting counterproductive work behavior), there are some positive implications for followers as well concerning their career progress. Again, findings point to the importance of moderating factors either on the side of the leaders (humility, constructive or destructive narcissism) or on the side of their followers (enactment behavior).

To begin with, Wisse et al. (2015) researched leaders' and followers' dark triad traits in relation to innovative behavior (idea generation, idea promotion, and idea implementation). In 306 dyads, leader narcissism moderated the positive relationship between followers' narcissism and their innovative behavior. The relationship did not occur for narcissistic leaders. The authors concluded that in hierarchical relationships, narcissism of the more powerful person will undermine the potential positive effects of narcissism on innovativeness at lower levels of the hierarchy.

Braun et al. (2016) researched leaders as well as followers as rating sources of leader narcissism and its outcomes in a multiple study series. Outcomes of leader narcissism included followers' emotions (malicious envy and benign envy) and counterproductive work behaviors directed against their leader. In response to narcissistic leaders, participants were more likely to experience malicious envy and counterproductive tendencies. These relationships were consistent across rating sources. However, the proposed negative relationship between leader narcissism and a more positive form of envy was inconsistent. In some studies, the authors found a weak negative relationship, while in others no significant relationships between leader narcissism and benign envy occurred. In another multisource survey design, Martin et al. (2016) collected data from 1,510 soldiers in the US army and 1,241 nominated followers. The final sample included 229 data sets with matching ratings from leaders, followers, and archival data. The authors found that parental income levels positively predicted self-rated leader narcissism, low engagement in desirable leader behaviors (i.e., task-, relational-, and change-oriented behaviors), which in turn negatively predicted perceived leadership effectiveness and followers' citizenship behaviors, and positively predicted followers' counterproductive behaviors.

Assessed from the followers' perspective, however, perceptions of leader narcissism appeared to be positively related to objective indicators of followers' career success in a sample of 811 employees in Germany (Volmer et al., 2016). Yet, in contrast to theoretical predictions, perceptions of leader narcissism did not relate significantly to followers' emotional exhaustion or job satisfaction.

Sosik et al. (2014) differentiated between two types of narcissism, constructive and destructive, in the relationship between leader charisma and followers' empowerment as well as their subsequent moral identity. As predicted, the relationship between charisma and empowerment was stronger when constructive narcissism was high and destructive narcissism was low. Moreover, the indirect effect of leader charisma on followers' moral identity was only significant for leaders with high constructive and low destructive narcissism.

In another attempt to reconcile the mixed effects of leader narcissism in leader-follower relationships, Owens et al. (2015) suggested that leaders' humility may “temper the potential negative effects of narcissism and magnify the potential positive effects” (p. 1204). In a sample of 876 employees rating 138 leaders from a Fortune 100 US health insurance organization, the interaction between leader narcissism and leader humility positively predicted four outcomes: perceived leader effectiveness, followers' job engagement, subjective and objective job performance. Leader narcissism predicted these outcomes only when leaders' humility was high.

Hochwarter and Thompson (2012) considered the role of followers' own behavior in response to leader narcissism. They suggested that in the face of narcissistic leaders, follower should “establish a level of personal control that minimizes present and future resource loss” (p. 339), so-called enactment behaviors. Findings supported this assumption in an investigation with three study samples. Followers' frustration and tension on the job increased with perceptions of their leaders' narcissism, but only for followers who displayed low enactment behaviors. Similarly, followers' perceptions of available resources and their own job performance decreased only for low-enactment followers.

To summarize, two original articles suggest that leader narcissism negatively impacts follower outcomes (emotions, counter productivity, citizenship), while one original article demonstrated a positive relationship with followers' career progress. The other three original articles point to the importance of moderating factors in the relationship between leader narcissism and follower outcomes at the dyadic level, including how narcissism is defined and measured, leaders' attitudes (e.g., humility), and followers' responses in the face of leader narcissism (e.g., enactment).

#### Team level of analysis

As compared to outcomes measured in leader-follower dyads, the empirical evidence of outcomes in work teams or small groups in response to leader narcissism is relatively scarce. One advantage of these often sophisticated studies is that they provide insights into causal relationships as experimental research designs are employed. They generally point toward the impact of leader narcissism on leadership emergence in teams. While narcissistic individuals are likely to emerge as leaders in teams, narcissistic leadership generally runs counter to successful group performance and positive interpersonal relationships over time.

Brunell et al. (2008) tested whether leader narcissism related positively to leader emergence in leaderless group discussions. Narcissistic group members were more likely to be seen as group leaders by themselves and others, and also described themselves as more motivated to lead. However, they were not more likely to achieve their goals in the group discussions, and hence did not perform better than less narcissistic group members. In addition, Ong et al. (2016) tested the notion that narcissistic individuals emerge as leaders in teams, but that leadership perceptions would deteriorate over time. Indeed, in teams of previously unacquainted and acquainted students, initial ratings of narcissism correlated with leadership ascribed by other team members. Twelve weeks later, however, the relationship was no longer significant. The authors conclude that teams “soon tire of leaders who display narcissistic traits” (p. 242).

Nevicka and colleagues provided findings on the impact of leader narcissism in team settings based on experimental research designs. While some of the results are based on student samples rather than field data from organizations, they provide initial insights into the dynamics that leader narcissism can enfold at the team level of analysis. Nevicka et al. (2011a) provided initial empirical evidence for the beneficial effects of narcissism on leadership emergence and leaders' own task performance in a team context. While individuals with high narcissism were generally more likely to be seen as leaders by others, reward structures influenced the extent to which narcissistic individuals contributed to their teams' successes. They showed higher performance when reward structures were interdependent (i.e., the best team was rewarded jointly) rather than when they were independent (i.e., the best players were rewarded individually). The researchers interpret this finding in the light of narcissistic strivings for recognition and external validation. In essence, interdependent rewards provide narcissists with a more attractive “stage to shine” in front of other team members (Nevicka et al., 2011a). Nevicka et al. (2011b), however, demonstrated that leader narcissism differentially affects perceptions of leaders and the actual behaviors of team members. While positive relationships occurred between team perceptions of leader narcissism, leader authority and leadership effectiveness, teams with narcissistic leaders performed worse in a joint task. They were less likely to share task-relevant information and made decisions of lower quality than teams with less narcissistic leaders.

Notably, findings by Wisse and Sleebos (2016) do not support explicit negative implications of narcissism in teams. The authors surveyed 225 supervisors and their 740 subordinates in Dutch organizations. They argued that leader narcissism reflects the comparably brighter side of the dark triad than Machiavellianism or psychopathy. Accordingly, in this study, leader Machiavellianism and psychopathy, but not leaders' narcissism positively predicted team members' perceptions of abusive supervision. The negative impact of Machiavellianism further increased when leaders felt they had high position power, that is, control over valued resources.

Taken together, results from these four original articles suggest that narcissists are not necessarily seen as bad leaders in team settings. However, the perceptions, which team members develop, may deteriorate over time. Moreover, the impact of leader narcissism on performance appears to be negative. While leader narcissism can fuel narcissistic individuals' performance under certain conditions (i.e., when performance opens up opportunities for external affirmation), it appears to hinder collaboration in teams.

#### Organizational level of analysis

##### CEO outcomes

Research that analyzes outcomes of leader narcissism at an organizational level has focused on top executives, especially Chief Executive Officers (CEOs). Narcissistic CEOs themselves appear to profit from their egocentric and bold decision-making. O'Reilly et al. (2014) collected ratings of narcissism for the CEOs of 32 US firms in the high-tech sector from 250 current employees. As expected, their findings demonstrated positive relationships between CEO narcissism and several indicators of executive compensation depending on the CEO's tenure. Long-tenured, narcissistic CEOs received the highest levels of compensation (measured via salary, bonus, stock options), held higher shares in the company (US$512 million more than less narcissistic CEOs, US$649 million more than their lower-tenured CEOs), and earned significantly more than members of their top management teams (US$5.1 million on average).

In contrast, narcissistic CEOs seem to care less about the constituents they lead. Focusing on the relations between CEO personality, leadership styles and strategic outcomes, Resick et al. (2009) studied a sample of 75 CEOs of Major League Baseball organizations over a 100-year period. The authors did not find the hypothesized negative relationship between CEO narcissism and transformational leadership, but CEO's core self-evaluations positively predicted their transformational leadership style. Yet, a predicted negative relationship between CEO narcissism and contingent reward leadership occurred. Thus, CEOs' positive self-concept predicts desirable leader behavior, while narcissism prevents it.

Supporting this view, in a study of 126 CEOs in the United States, self-ratings of narcissism correlated negatively with servant leadership (rated by Chief Financial Officers), an other-oriented form of leadership (Peterson et al., 2012). The relationship was mediated through CEO's organizational identification. Hence, narcissistic CEOs identified less with the company and were less likely to be seen as other-oriented leaders. Even though the authors did not propose a direct link between CEO narcissism and firm performance, CEO's servant leadership in turn was positively related to the company's return on assets.

In sum, findings from these three original articles suggest that narcissism benefits idiosyncratic gains on the side of the leader, but runs counter to leader behavior that would be beneficial to the constituents they lead within their organizations.

##### Organizational strategy and culture

In Chatterjee and Hambrick's (2007) seminal study, indicators of narcissism early in the CEO's tenure predicted organizational outcomes in subsequent years including more strategic dynamism. A dynamic strategy was reflected by changes in resource deployment, number and size of acquisitions by the company, extreme performance in total shareholder returns (TSR), and fluctuation of performance in the form of return on assets (ROA). Even though results partly varied across different indicators for each of the outcome variables, this research provided considerable insights into the implications of nominating a narcissistic CEO for organizational strategy. It also inspired many following studies based on the same measure of CEO narcissism.

Patel and Cooper (2014) analyzed the impact of CEO narcissism on company performance both during and after an economic crisis. Building on the idea that narcissists should be approach motivated (i.e., driving wins), but not avoidance motivated (i.e., inattention to losses), the authors predicted positive relations between CEO narcissism and post-crisis company performance, but negative relations between CEO narcissism and company performance at crisis onset. In line with these predictions, CEO narcissism was negatively related to returns at the start of a crisis period and positively related to returns in the post-crisis period.

Aktas et al. (2016) analyzed narcissism of both the acquiring company's and the target company's CEO in the context of mergers and acquisitions (M&A). They coded narcissism through the prevalence of first-person singular pronouns in 1,780 interviews for a sample of 146 M&A deals. Acquirer CEO narcissism predicted initiation of the takeover process, shorter process length, and lower probability for the target CEO to obtain a prestigious position in the merged firm. When both acquirer and target CEO were highly narcissistic the probability of deal completion decreased.

Oesterle et al. (2016) found for the 31 largest German manufacturing firms that CEO narcissism predicted growth in general internationalization strategies (ratio of foreign sales to total sales). The authors conclude that international growth opens up opportunities to fulfill CEOs' narcissistic interests by raising their sphere of control. However, as opposed to the authors' predictions, CEO narcissism did not increase the share of foreign sales with high risk, that is, in countries with high psychic distance to the home market (in this case, Asia).

Jones et al. (2004) conducted a case study analysis of a business organization operating in Australia and Southeast Asia. Data stemmed from 24 months of observation, resulting in a comprehensive journal with observation notes, personal interpretations and inferences drawn by the researcher. They were complemented with insights from semi-structured interviews with the CEO and other managerial and non-managerial staff. The authors linked the CEO's narcissistic values and behaviors (e.g., striving for affirmation, demanding obedience) to the development of a strong dominant culture. Unclear mission and goals, rewarding loyalty and commitment above performance, a rigid view of trust as the result of long tenure, and superficial happiness characterized this culture. At the same time, the authors found “a powerful counterculture comprised of professional managerial staff who hold very different values and assumptions” (Jones et al., 2004, p. 227) and are driven by clear achievable goals. Nevertheless, the narcissistic CEO maintains a “climate of fear, compliance and subversion of individual thought and willpower” (Jones et al., 2004, p. 227), creating clear distinctions between the company's in-group and out-group.

In sum, results from these five original articles suggest that leader narcissism relates to risk-taking in organizational strategy (e.g., initiating M&A deals, expanding into new markets) and a problematic organizational culture. Narcissistic CEOs approach opportunities for gains, yet are less attuned to recognizing potential losses. Their approach to strategy seems to be driven by a strong in-group orientation, but they lack clear goals and objectives. Organizations with narcissistic CEOs are therefore more likely to fluctuate in their performance than organizations led by their less narcissistic counterparts.

##### Entrepreneurial orientation

Wales et al. (2013) found a positive relationship between self-ratings of CEO narcissism and variations of company performance in 173 US based firms of small and medium size (10–250 employees) and young in age (10 years or less). The relationship was mediated by entrepreneurial orientation, that is, organizational strategy-making policies and practices to identify and launch new ventures (manifested in innovativeness, risk-taking, proactiveness). Narcissistic CEOs rated entrepreneurial orientation of their firms higher than their less narcissistic counterparts, which in turn predicted higher variations in companies' sales performance

In a recent study, Engelen et al. (2016) further differentiated market conditions, which can alter the impact of CEO narcissism on company performance in an entrepreneurial context. In a sample of 41 companies from the U.S. high-tech sector, entrepreneurial orientation positively predicted shareholder value. In firms with highly narcissistic CEOs, shareholders generally profited less from the company's entrepreneurial orientation. Yet, the impact of CEO narcissism also depended on market conditions. In highly concentrated and in dynamic markets, the relationship between entrepreneurial orientation and shareholder value increased with CEO narcissism. It appears that under certain contextual conditions, narcissistic CEOs' bold actions, such as risk-taking or trial-and-error strategies, can be effective.

Gerstner et al. (2015) corroborated further evidence supporting this notion, studying strategic investment initiatives in biotechnology made by 72 CEOs of 33 US pharmaceutical firms. Again, narcissistic CEOs seemed more prone to take bold actions, making more investments and directing the attention of their managerial team to new technology. The strength of these relationships increased for technologies that received greater attention from the public. Narcissistic CEOs may anticipate public admiration for these investments in the future, and hence perceive them as one means to satisfy their need for external attention and recognition. The authors conclude that, in contrast to their less narcissistic counterparts, “narcissist's supreme confidence allows him or her to invest aggressively” (p. 279).

Overall, these three original articles suggest that CEO narcissism aligns with an entrepreneurial orientation. Narcissistic CEOs are open to entrepreneurial actions, such as investment into new technologies. However, whether this entrepreneurial orientation benefits the organization and its stakeholders depends on the market conditions. Initial evidence suggests that CEO narcissism is more beneficial in markets that require bold or change oriented action.

##### Organizational image

One concern with narcissistic leaders is that their actions are outward oriented, but neglect the interests and needs of internal stakeholders. Accordingly, narcissistic CEOs may engage in “window dressing” activities, which enhance an organization's external image, but not necessarily its performance. In line with this view, Petrenko et al. (2016) suggested that narcissism drives CEOs to engage in corporate social responsibility (CSR) practices “as a way to enhance their moral feelings of superiority and to attract attention and praise” (p. 263). As predicted, CEO narcissism correlated positively with external CSR evaluations. CEO narcissism also contributed to increases in corporate philanthropy media profiles. In contrast, higher levels of CEO narcissism weakened the relationship between corporate CSR and firm performance. The authors interpret this finding to suggest that narcissistic CEOs mainly engage in CSR practices for purposes of personal need fulfillment and image reinforcement, but do not align these actions with strategies that would benefit corporate success more generally.

Similarly, research in the context of corporate accounting tested the notion that CEOs with high levels of narcissism may be motivated to make accounting decisions that improve the external financial appearance of their company. Olsen et al. (2014) observed positive relationships of CEO narcissism with earnings-per-share (EPS) and stock prices, both of which are public financial performance measures (and thus would be likely to serve narcissistic CEO's needs for external affirmation). However, when testing several mediating effects, the authors found that narcissistic CEOs' operational decisions (e.g., lenient credit terms, sales discounts, over production) drove EPS rather than accounting related decisions (e.g., restatement). In contrast, Olsen and Stekelberg (2016) reported relationships between CEO narcissism and tax-sheltering practices based on measures of uncertain tax benefits (positively predicted by CEO narcissism) and cash effective tax rates (negatively predicted by CEO narcissism). These results align with findings by Rijsenbilt and Commandeur (2013) indicating a positive relationship between an objective index of CEO narcissism and fraud accusations in sample of 54 CEOs and 113 Accounting and Auditing Enforcement Release allegations.

Further evidence from two simulation studies took into account how external factors interact with CEO narcissism to predict financial misreporting. Chen (2010a) conducted a computer simulation including the variables CEO dishonesty, CEO narcissism, shareholder expectations, and media praise. The study used a system dynamics approach to model how these factors interact in self-reinforcing loops. CEO dishonesty and narcissism were seen to interact and predict an exponential growth pattern for misreporting, which was higher than for CEO narcissism alone. However, the steepest rise in financial misreporting occurred for the additional assumption that shareholders held increasing expectations toward company performance (i.e., outperforming the industry average as the norm), and when media praise positively reinforced the effects of CEO narcissism as well as shareholder expectations. Chen (2010b) extended the simulations by several dampening factors, predicting that social constraints on CEOs' self-aggrandizement (e.g., through national culture) restrict the influence of CEO narcissism on misreporting. The simulation results of both studies suggested a vicious circle in which positive media commentaries and rising shareholder expectations fueled the impact of CEOs' narcissistic tendencies on financial misreporting.

In summary, these six original articles provided a mixed picture of the impact that CEO narcissism may have on an organization's external reputation. Narcissism appeared to drive a motivation for “window dressing,” that is, engaging in CSR activities without targeting sustainable outcomes of these activities. Two studies suggested a relationship between CEO narcissism and activities that may be interpreted as fraudulent, while one study indicated that legal operational decisions are taken to raise the companies' external image.

##### Top management team

Relatively little is known about the interactions between narcissistic CEOs and their top management teams. Drawing from individual and team levels of analysis, one would expect that narcissistic CEOs also lack the skills to motivate and guide successful teamwork at the top management level. However, findings by Reina et al. (2014) only partly supported this notion. The authors analyzed a sample of 97 CEOs from companies in the US computer software and hardware industry. CEOs' organizational identification moderated the relationship between narcissism and top management team (TMT) behavioral integration and firm performance. For CEOs whose identification with the organization was low, narcissism related negatively to integrated TMT behaviors (collaborative behavior, information exchange, joint decision making) and the companies' return on assets. The opposite was the case for CEOs with high organizational identification, where results demonstrated positive relationships between CEO narcissism and the two outcomes.

Initial empirical evidence also points to the influence of leader narcissism on top management team compilation. Since narcissists strive for external affirmation, they should be prone to hire team members with high similarity in terms of personal values and attitudes. Zhu and Chen (2015) found for a sample of 292 companies from the Fortune 500 list that CEOs tended to hire directors with levels of narcissism similar to their own. Similarity in narcissism as well as directors' previous experience with CEOs of similar narcissism levels in turn predicted CEOs' risk-taking spending. The authors concluded that similarity as well as previous experience rendered directors more supportive of their CEOs' risk-taking behaviors.

Hence, these initial results suggest that CEO narcissism can hinder collaboration in top management teams, but that this depends on the relevance that CEOs attach to their firm. Furthermore, narcissistic CEOs appear to shape personnel decisions in ways that provide them with lower restrictions from other executives within the firm.

## Gaps in research and implications

In the following, we build on the above-presented research and discuss findings on the positive as well as negative outcomes of leader narcissism. Next, we turn to demonstrate the need for further research of leader narcissism in relation to its outcomes at multiple levels of analysis in organizations. The discussion starts by highlighting inconsistencies of results within as well as across levels. The subsequent parts then derive implications for stronger theory building as well as necessary methodological advancements. Finally, we acknowledge the limitations of this review and provide a brief outlook for the research of narcissism and leadership in organizations.

### Summary

#### Positive outcomes of leader narcissism

The reviewed original articles suggest that there are few positive outcomes of leader narcissism across levels of analysis. Narcissistic leaders are more likely to be seen as charismatic figures than their less narcissistic counterparts with the skills to communicate bold and daring visions (Galvin et al., 2010). These findings from the dyadic level of analysis correspond to research assessing outcomes for the entire organization. Narcissistic CEOs act in forward driven manners, for example, they are more likely to initiate Mergers & Acquisitions (Aktas et al., 2016), invest in internationalization (Oesterle et al., 2016) or new technology (Gerstner et al., 2015). Narcissistic leaders also appear to perform well when given opportunities for external affirmation (Nevicka et al., 2011a) or to further their idiosyncratic gains (e.g., CEO pay and bonuses; O'Reilly et al., 2014). According to initial evidence, leader narcissism may contribute to followers' career building (Volmer et al., 2016). Yet, as will be discussed below, further research is needed to explore the conditions of this potentially positive dynamic.

Generally, the dynamics that leader narcissism enfolds in organizations require some caution (Chatterjee and Hambrick, 2007). Depending on contextual conditions, leader narcissism appears to be more or less fit for purpose. Findings at the dyadic level suggest that followers prefer narcissistic leaders when they feel uncertain (Nevicka et al., 2013). Similarly, organizations seem to profit from narcissistic CEOs under dynamic market conditions (Engelen et al., 2016). They follow a general approach strategy, but lack the sensitivity to adapt this strategy to external conditions (e.g., avoiding losses at the onset of crisis; Patel and Cooper, 2014).

#### Negative outcomes of leader narcissism

The literature review suggests many negative consequences of leader narcissism for organizations. First and foremost, narcissists lack concern for others. They see themselves as transformational leaders, but this view does not seem to be reciprocated by others. Findings confirm the misfit between how narcissists view their own leadership qualities and the impressions of others around them at the dyadic level (Judge et al., 2006; Greaves et al., 2014) as well as for narcissistic CEOs (Resick et al., 2009; Peterson et al., 2012). This apparent lack of self-awareness in narcissistic leaders is particularly troublesome as earlier research demonstrates the negative impact of over-estimation in leaders' self-evaluations (Atwater and Yammarino, 1992; Atwater et al., 1998).

Second, initial evidence suggests negative consequences of leader narcissism for followers' emotions (envy; Braun et al., 2016) and behaviors (e.g., counterproductivity and citizenship; Martin et al., 2016) at the dyadic level. These findings support the notion that future research must further explore the dynamics of negative emotions and behaviors triggered by narcissistic leaders. Leader narcissism hinders fruitful collaboration in teams (Nevicka et al., 2011b), a finding that is mirrored also by results at the top management team level (Reina et al., 2014). Negative forms of leadership cause emotional and behavioral downward spirals in organizations (e.g., through upward and downward revenge; Kim et al., 1998). Yet, we know very little about the spread of negative emotions or behaviors in teams in response to leader narcissism.

Finally, organizational-level outcomes indicate that narcissistic CEOs engage in unsustainable, “window dressing” activities (e.g., CSR; Petrenko et al., 2016). Through these activities, tailored to better the external image in the short run, narcissistic leaders put the organization's reputation at risk. They may even go so far as to commit fraud (Rijsenbilt and Commandeur, 2013; Olsen and Stekelberg, 2016). These findings provide a compelling case for the importance of further research into leader narcissism. We argue below that not only the consequences of leader narcissism must be explored, but also their moderating conditions and the effectiveness of interventions require systematic testing.

### Gaps and suggestions for future research

#### Theoretical implications

Despite considerable insights that the reviewed research has provided, fundamental questions about leader narcissism and its impact on organizations remain unanswered. There is no conclusive answer to the question whether narcissists are effective leaders. According to previous results, the answer depends on rating sources (e.g., supervisors or subordinates; Judge et al., 2006), the effectiveness criterion (e.g., interpersonal or task performance; Blair et al., 2008), individual difference variables (e.g., leader and follower gender; De Hoogh et al., 2015), and contextual factors (e.g., ethical organizational practice; Hoffman et al., 2013). In addition, too little attention has been paid to the question how narcissistic leaders relate to other members of the organization, above and beyond their own subordinates. Future research can explore how they build relationships with other, less narcissistic peers in leadership positions. Another focus for future research would be how narcissists develop strategic networks to bolster their self-esteem and influence decisions.

Previous research remains ambiguous about the conceptualization and subsequent assessment of leader narcissism. While the majority of studies build on theoretical views of a multidimensional construct (e.g., the four dimensions suggested by Emmons, 1987), the measurement of leader narcissism rarely tested their differential impact. Only few authors differentiated between constructive and destructive narcissism (Sosik et al., 2014) or profiles of high bright-side and low dark-side characteristics (Paunonen et al., 2006). It remains unclear to what extent these conceptualizations are theoretically valid and adequately reflect the construct of narcissism. Miller et al. (2017) suggest that controversies around narcissism stem from the unclear distinctions between its grandiose and vulnerable forms. They advocate a unified construct with central features that are shared across the two forms (i.e., interpersonal antagonism) and peripheral ones that distinguish the two (i.e., neuroticism for vulnerable narcissism and agentic extraversion for grandiose narcissism). The majority of studies also considered leader narcissism in isolation rather than relative to other positive or negative traits of a leader. For example, Wisse and Sleebos (2016) found Machiavellianism, but not narcissism or psychopathy to predict perceptions of abusive supervision. Results by Owens et al. (2015) indicated leaders' humility to moderate the relationship between leader narcissism and outcomes. Building on these inconsistencies and gaps in the current research, in the following we suggest three theoretical pathways toward an advanced understanding of leader narcissism in organizations.

First, future empirical research of leader narcissism must consider existing theory more carefully. Theories such as the dynamic self-regulation model of narcissism (Morf and Rhodewalt, 2001) enable more nuanced views than an overall, one-dimensional narcissism construct. A stronger theoretical basis will also provide researchers with clearer guidelines whether they might test non-linear rather than linear relationships between leader narcissism and its outcomes (Grijalva et al., 2015). Future research can also help to differentiate between types or “flavors” of narcissism and allow testing nuanced profiles of narcissistic personalities (e.g., low self-esteem combined with high or low impression management). Detecting these nuances will require in-depth exploration through interviews, case studies or other qualitative methods. Future studies need to look at vulnerable as compared to grandiose forms of narcissism in leaders. We do not know whether vulnerable narcissists simply do not seek out opportunities for leadership or whether their motivations to lead differ from those of individuals with high levels of grandiose narcissism (Chan and Drasgow, 2001). Alternatively, grandiose narcissists might be motivated to lead, while vulnerable narcissists are more likely to follow (Kark and Van Dijk, 2007). Benson et al. (2016) studied narcissists' reactions when they were assigned to followership roles, demonstrating that they perceived these roles poorly and did not occupy them well (e.g., high self-interest and low willingness to act for the benefit of the collective). We encourage research to clarify these relations, using advanced measures for this purpose (e.g., Pathological Narcissism Inventory, PNI; Pincus et al., 2009), which would also contribute to a better integration of leadership and followership theory.

Second, future studies need to advance hypotheses that explicitly connect leader narcissism to its consequences at multiple levels of analysis in organizations. In the research reviewed here, several similarities occurred across levels (e.g., leader narcissism as a positive predictor of risk-taking). However, none of the studies tested the impact of leader narcissism at multiple levels of analysis simultaneously. We propose person-environment (PE) fit as a useful theoretical lens in this regard. PE fit concerns “the compatibility between an individual and a work environment that occurs when their characteristics are well matched” (Kristof-Brown et al., 2005, p. 281). Fit can be based on multiple elements, including one's fit with the organization, one's team, a specific job or a vocation (Edwards, 1991; Cable and DeRue, 2002). The literature reviewed here suggests that leader narcissism can have positive consequences, but only under certain conditions (e.g., dynamic markets). In other words, narcissistic leaders might be more “fit for purpose” in some environments than in others. We assert that future work can assess the validity of these findings through advanced theorizing. For example, in industries where self-aggrandizing presentation is fit for purpose (e.g., marketing/social media) narcissistic leaders may generate positive outcomes at the firm level (e.g., sales, return on assets), but still antagonize fruitful collaboration in teams or with individual followers. Understanding the conditions under which leader narcissism harms or bolsters organizational functioning at multiple levels will inform recommendations for practice.

Finally, especially given initial results provided by Ong et al. (2016), future research needs to better understand the impact of leader narcissism over time. Their findings suggest that relationships between narcissistic leaders and their followers are likely to deteriorate over a relatively short period of time (12 weeks). Narcissistic leaders are likely to hold positions in organizations over several years, but we do not know exactly, at which point in time their negative or positive impact enfolds (Shamir, 2011). This knowledge will also be necessary to develop and test interventions that target the negative consequences of leader narcissism.

**Figure 1:**
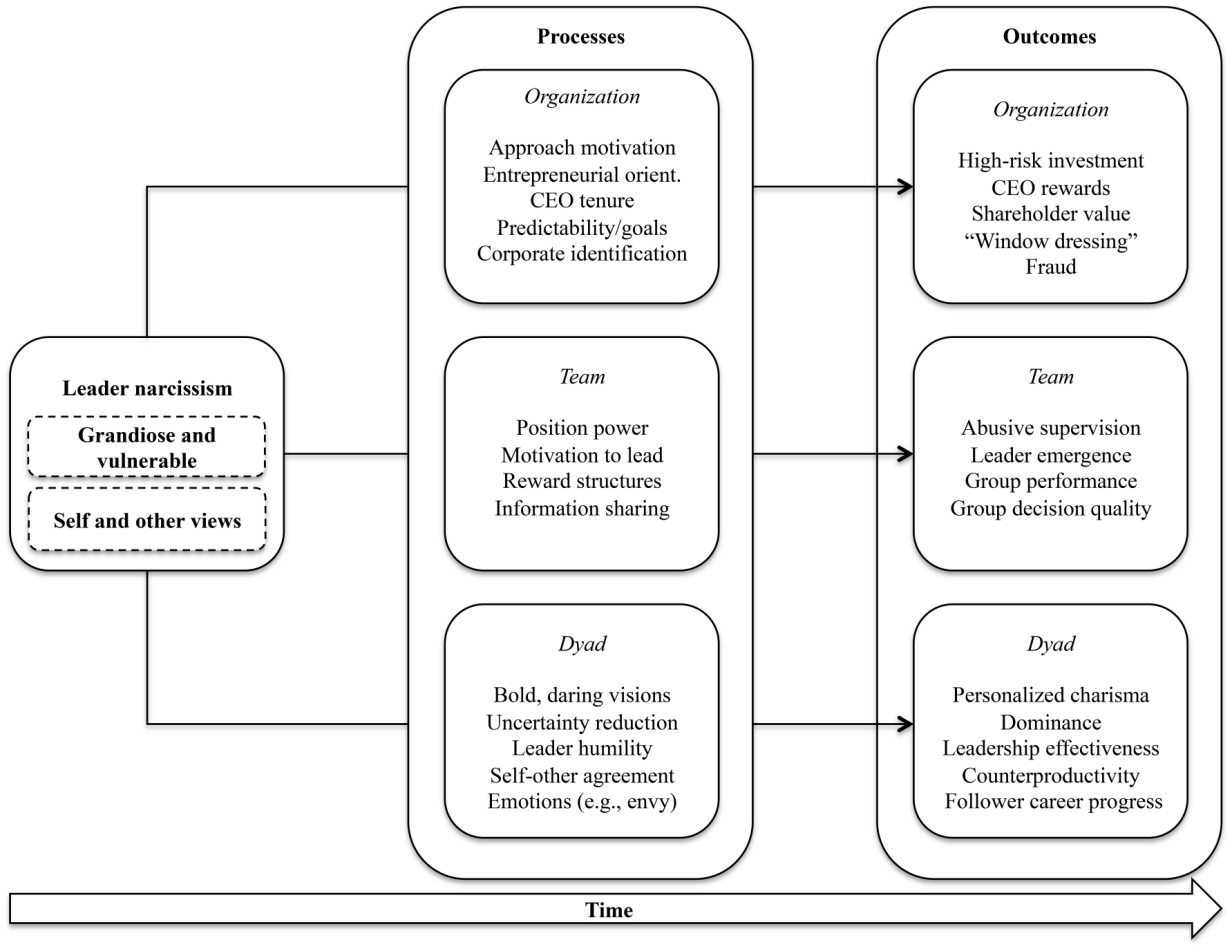
**Summary of findings**.

Figure 1 summarizes the main insights gained from this review and provides a model of the relationships between leader narcissism and outcomes at multiple levels of analysis in organizations. The model serves to inform and guide future research in the field.

#### Methodological implications

The results presented in this review suggest that research of leader narcissism in organizations will profit from several methodological advancements. The majority of studies of leader narcissism to date have provided insights based on field data and cross-sectional research designs (with notable exceptions: Nevicka et al., 2011a,b, 2013; Braun et al., 2016). The field approach enhances external validity, but often does not allow for causal conclusions about the variables of interest or deeper insights into the underlying intrapersonal and interpersonal dynamics. We firstly recommend the use of experimental research designs, more frequently applied in the social sciences, to test the causal mechanisms through which leader narcissism impacts outcomes in organizations (Aguinis and Bradley, 2014). For example, this approach would allow researchers to systematically induce variations of state self-esteem and test the extent to which grandiose or vulnerable narcissists respond to these variations with abusive supervisory behavior (Wisse and Sleebos, 2016). Secondly, we strongly advocate the use of innovative quantitative and qualitative research designs. For example, case studies help understand the dynamics that one narcissistic leader can enfold across an entire organization as well as the subtle responses of different groups of followers to these dynamics (Jones et al., 2004). Computer simulations are a relatively novel approach in the social sciences. They can inform our understanding of how leader narcissism interacts with different organizational variables (e.g., formal regulation, punishment of misconduct), and how changing them can alter the impact of leader narcissism on subsequent outcomes (Chen, 2010a,b). Also analyzing historical developments in relation to leadership in the political realm informs the current understanding of how narcissistic leaders influence their audiences (Deluga, 1997).

In addition, given that previous research shows variations between self and other ratings of narcissism (Judge et al., 2006; Braun et al., 2016; Volmer et al., 2016), we advocate for a stronger theoretical rationale why one or the other is used in a given study. While self-ratings of narcissism measure the individual's personality trait, external ratings seem to better reflect a behavioral style (e.g., narcissistic leadership; Rosenthal and Pittinsky, 2006). Recent research also suggests that a form of state narcissism can be differentiated from the original trait (Giacomin and Jordan, 2016). Individuals can down-regulate their state narcissism through interventions that increase their communal focus (Giacomin and Jordan, 2014). We advocate two advancements in this regard, that is, using event based sampling techniques to collect data of leader narcissism as a state variable (e.g., daily diary methods; Ohly et al., 2010) and testing short and long term interventions to counteract the negative impact of leader narcissism.

### Limitations

This review of the literature is not without limitations, which must be taken into account when interpreting the above stated insights. Firstly, we selected a specific set of studies in line with the focus of this review, which at the same time led to the exclusion of others. For example, research in the field of narcissism has been conducted from a psychoanalytical perspective (e.g., Gabriel, 1997). We acknowledge the value of this work for the concept of narcissism generally. However, the purpose of this review was to address how leader narcissism relates to outcomes at dyadic, team, and organizational levels of analysis from an organizational psychology perspective.

Secondly, we included only original articles published in scholarly, peer-reviewed journals. This approach concurs with previous work suggesting that peer review ensures theoretical and empirical rigor of research included in a review (Gardner et al., 2011). We acknowledge that on the downside, this decision can bias conclusions drawn from this review in the light of the file drawer problem (Rosenthal, 1979; McDaniel et al., 2006; Howard et al., 2009).

Thirdly, we acknowledge the significant insights that meta-analyses have provided into the relations between narcissism and organizational outcomes (O'Boyle et al., 2012) as well as narcissism and leadership (Grijalva et al., 2015). We decided to approach the question of this article through a systematic literature review, and thereby to integrate findings from different forms of assessment of narcissism at multiple levels of analysis through a qualitative lens. While this approach enabled us to integrate findings across different study methodologies (e.g., field surveys, laboratory experiments, case studies, computer simulations), we acknowledge the limitations of this decision. We did not compare the strength of relationships and effect sizes in a standardized manner across studies or for the specific measurement scales used. Nevertheless, insights provided by this review are sought to inspire theory building and methodological advances in the study of leader narcissism and its outcomes. Future research can build on these advances and analyze subsequent study results with meta-analytical procedures.

## Conclusion

Organizations seem to have turned into a “me-me-me” world of narcissism. Scholars and the public are therefore concerned with the negative impact that narcissism may enfold, especially when narcissists gain leadership positions. In the face of increasing empirical insights, this systematic literature review sought to present a nuanced view of what we know so far about the negative, but also positive consequences of narcissism in organizations. The insights presented speak in favor of a more balanced picture, taking into account different levels of analysis in organizations and surrounding conditions, under which leader narcissism can enfold its dynamics. The hope of this review is to inspire fruitful theory building as well as methodological advancements in the field of narcissism and leadership.

## Author contributions

The author confirms being the sole contributor of this work and approved it for publication.

## Funding

I am immensely grateful to Dr. David Chivers, Dr. Ellen Schmid and Professor Barbara Wisse for their helpful comments on earlier versions of this paper. I would also like to express my gratitude to the Institute of Advanced Study (IAS) at Durham University. The Sir Derman Christopherson/Sir James Knott Foundation Fellowship 2016-17, administrated by the IAS, allowed me to work on this review. I sincerely thank the IAS Directorate and Fellows for the interdisciplinary discourse that furthered this work.

### Conflict of interest statement

The author declares that the research was conducted in the absence of any commercial or financial relationships that could be construed as a potential conflict of interest.
